# Anti-VEGF therapy resistance in ovarian cancer is caused by GM-CSF-induced myeloid-derived suppressor cell recruitment

**DOI:** 10.1038/s41416-019-0725-x

**Published:** 2020-01-14

**Authors:** Naoki Horikawa, Kaoru Abiko, Noriomi Matsumura, Tsukasa Baba, Junzo Hamanishi, Ken Yamaguchi, Ryusuke Murakami, Mana Taki, Masayo Ukita, Yuko Hosoe, Masafumi Koshiyama, Ikuo Konishi, Masaki Mandai

**Affiliations:** 1 Department of Gynecology and Obstetrics, Kyoto University Graduate School of Medicine, Kyoto, Japan; 2 Department of Obstetrics and Gynecology, National Hospital Organization, Kyoto medical center, Kyoto, Japan; 3 Department of Obstetrics and Gynecology, Kindai University Faculty of Medicine, Osaka, Japan; 4 Department of Obstetrics and Gynecology, Iwate Medical University School of Medicine, Iwate, Japan; 5 The University of Shiga Prefecture, Shiga, Japan

**Keywords:** Ovarian cancer, Chemokines

## Abstract

**Background:**

The mechanism of resistance development to anti-VEGF therapy in ovarian cancer is unclear. We focused on the changes in tumour immunity post anti-VEGF therapy.

**Methods:**

The frequencies of immune cell populations and hypoxic conditions in the resistant murine tumours and clinical samples were examined. The expression profiles of both the proteins and genes in the resistant tumours were analysed. The impact of granulocyte–monocyte colony-stimulating factor (GM-CSF) expression on myeloid-derived suppressor cell (MDSC) function in the resistant tumours was evaluated.

**Results:**

We found a marked increase and reduction in the number of Gr-1 + MDSCs and CD8 + lymphocytes in the resistant tumour, and the MDSCs preferentially infiltrated the hypoxic region. Protein array analysis showed upregulation of GM-CSF post anti-VEGF therapy. GM-CSF promoted migration and differentiation of MDSCs, which inhibited the CD8 + lymphocyte proliferation. Anti-GM-CSF therapy improved the anti-VEGF therapy efficacy, which reduced the infiltrating MDSCs and increased CD8 + lymphocytes. In immunohistochemical analysis of clinical samples, GM-CSF expression and MDSC infiltration was enhanced in the bevacizumab-resistant case.

**Conclusions:**

The anti-VEGF therapy induces tumour hypoxia and GM-CSF expression, which recruits MDSCs and inhibits tumour immunity. Targeting the GM-CSF could help overcome the anti-VEGF therapy resistance in ovarian cancers.

## Background

Ovarian cancers represent the most lethal group of gynaecological malignancies, as most patients are diagnosed at an advanced stage.^1^ Despite advancements in chemotherapy and surgery, patients’ prognoses have not significantly improved over the last few decades.^2^ Bevacizumab, an anti-VEGF antibody and an anti-angiogenic agent, was approved as the first effective molecular therapeutic against ovarian cancer. Bevacizumab in a combination with standard cytotoxic chemotherapies and subsequently, for maintenance monotherapy, prolongs progression-free survival of advanced ovarian cancer patients.^3^ Although few cases achieve complete cancer clearance, most cases show a relapse during or after bevacizumab treatment.^4^ Bevacizumab reduces ascites effectively. However, the single agent is only modestly effective on suppressing tumour progression. Therefore, elucidating the mechanism of resistance to anti-VEGF therapy in ovarian cancer and overcoming the same is urgently required.

The acquisition of immune evasion by tumour cells is one of the major mechanisms of tumour progression,^5^ and mainly consists of immunosuppressive cells in the tumour microenvironment and loss of immune checkpoint signalling. We previously showed that expression of programmed cell death ligand-1 (PD-L1), an immune-checkpoint molecule, was associated with patients’ poor prognoses in ovarian cancer.^6,7^ Anti-PD-L1 antibody proved to be effective against recurrent ovarian cancer cases with platinum resistance.^8^ However, PD-1/PD-L1 blockade exhibited durable response to only a small portion of platinum-resistant ovarian cancer cases. Therefore, the elucidation of the other immunosuppressive mechanisms is required. We also showed that an infiltration of MDSCs into ovarian cancer is associated with patients’ poor prognosis.^9^ MDSCs, which exhibit an immunosuppressive ability in the tumour microenvironment, are defined as a heterogeneous population comprising myeloid lineage cells at various stages of differentiation that expand from bone marrow into peripheral organs, including the tumour microenvironment.^10^ Several studies have shown that infiltration of MDSCs is positively associated with the drug resistance as well as the tumour progression.^11^ Various chemoattractants for MDSCs have been reported;^12^ however, the most significant inducer of MDSCs in ovarian cancers remains unclear.

In general, GM-CSF is an essential factor for the differentiation and maintenance of myeloid cell lineage in bone marrow.^13^ Some reports showed that GM-CSF promoted recruitment of MDSC in tumour microenvironment;^14,15^ however, other reports showed that GM-CSF activated T-cell response in tumour.^16^ Furthermore, GM-CSF was reported to directly promote tumour cell proliferation.^17^ The role of GM-CSF in tumour microenvironment remains unclear. Furthermore, there is no available report that associates GM-CSF with resistance to antitumour agents.

In this study, we show that hypoxia in tumours induced by anti-VEGF antibody treatment induces GM-CSF expression, which causes MDSC recruitment into tumour sites, leading to immunosuppression and tumour progression, and in turn, resistance to anti-VEGF therapy.

## Methods

### Cell lines and tumour models

The OV2944-HM-1 (HM-1) mouse ovarian cancer cell line was purchased from RIKEN BioResource Center on January 2003 and cultured as described.^7^ The ID8 mouse ovarian cancer cell line and its *Vegf-a*-overexpressing derivative (ID8-Vegf) were kindly provided by Dr Katherine Roby on September 2009. These cells were cultured and maintained in RPMI1640 (Invitrogen, Carlsbad, CA, USA) supplemented with 10% foetal bovine serum (FBS), 100 U/mL penicillin and 100 g/mL streptomycin in a 5% CO_2_ atmosphere at 37 °C. Those tumours were proved to have immunogenicity, and often used for immunologic research. Histologically, ID8 and ID8-Vegf cells imitate adenocarcinoma.^18^ HM-1 cells were previously shown to express a high level of VEGF and have invasive characteristics.^19^ Throughout the work, we used ID8, ID8-Vegf and HM-1 cell lines passaged fewer than 30 times. All cells were regularly tested for mycoplasma contamination. Authentication of these cells with short tandem repeat analysis was not performed because they were derived from mice.

Female C57BL/6 (B6) mice were purchased from CLEA Japan (Tokyo, Japan) and B6C3F1 (C57BL6 × C3/He F1) mice from Charles River Japan (Yokohama, Japan). Animal experiments were approved by the Kyoto University Animal Research Committee, and animals were maintained under specific pathogen-free conditions. A total of 5 × 10^6^ ID8-Vegf or ID8-GFP control cells were inoculated intraperitoneally into B6 mice, and 1 × 10^6^ HM-1 cells were inoculated subcutaneously into the right flank of B6C3F1 mice. Anti-VEGF antibody (Genentech, South San Francisco, USA, clone B20-4-1-1) and anti-GM-CSF antibody (Bioxcell, West Lebanon, USA, clone MP1-22E9) treatment were initiated 4 days after the tumour inoculation and administered intraperitoneally at 5 mg/kg/mouse and 100 μg/mouse twice a week for 24 days, respectively. B20-4-1-1 is reported to be a cross-species reactive, function-blocking mAb targeting both human and murine VEGF-A.^20^ CXCR2 antagonist treatment (Tocris, UK, clone SB265610) was administered intraperitoneally at 2 mg/kg/mouse five times a week for 24 days. Subcutaneous tumour size and body weight were measured twice a week, and tumour volumes were calculated as volume = LD × SD^2^ × 0.4, where LD is the long diameter (mm) and SD (mm) is the short diameter of the tumour. B6 mice bearing peritoneal dissemination were sacrificed by carbon dioxide euthanasia when they suffered from massive ascites.

### Immunohistochemistry of mouse tumours

Mouse tumour cryosections (6 μm) were stained with anti-CD8 (Abcam, clone YTS169.4), anti-Gr-1 (BD Pharmingen, clone RB6-8C5) and anti-CD31 (BD Bioscience, clone MEC 13.3) antibodies as described.^9^ Hypoxyprobe^TM^-1 OMNI kit (HPI Inc., Burlington, USA) was used for detection of hypoxic area in mouse tumour samples following the manufacturer's protocol. Image J software was used for quantification of CD31-positive area and pimonidazole-positive area in tumour. For immunofluorescence staining, anti-IgG labelled with AlexaFluor 488 (Abcam, Kenbridge, UK) and AlexaFluor 594 (Abcam) were used as secondary antibodies. Nuclei were stained with 4′,6-diamidino-2-phenylindole (DAPI) solution (Dojindo, Kumamoto, Japan). Fluorescence images were captured using a Keyence BZ-X700 microscope (Keyence, Osaka, Japan).

### Flow cytometry

Mice with tumour formation were euthanised by CO_2_ gas, and their spleens, femurs, tibias and tumours were collected. Cells were stained with the following antibodies (BioLegend, San Diego, USA) for 30 min at 4 °C: anti-mouse CD3 (clone 145-2C11), anti-mouse CD4 (clone RM4-5), anti-mouse CD8a (clone 53-6.7), anti-mouse CD45 (clone 30-F11), anti-mouse Gr-1 (clone RB6-8C5), anti-mouse CD11b (clone M1/70), anti-mouse Ly6G (clone 1A8), anti-mouse Ly6C (clone AL-21), anti-mouse F4/80 (clone BM8) and anti-mouse CD11c (clone N418). Non-viable cells were stained with 7-Amino-actinomycin D (AAD) staining solution or DAPI solution, or Zombie Aqua^TM^ and gated out. Matched isotype antibodies were used as controls. Data were acquired using a MACS Quant (Miltenyi Biotec, Bergisch Galdbach, Germany) and analysed by MACS Quantify (Miltenyi Biotec).

### Gene expression microarray analysis

HM-1 subcutaneous tumour specimens were collected after treatment with anti-VEGF antibodies or rat IgG control. The total RNA from the tissues was extracted by use of the RNeasy Mini Kit (QIAGEN, Venlo, The Netherlands). The total RNA expression was analysed by using the Mouse Transcriptome Assay 1.0 (Affymetrix, Santa Clara, CA, USA), and robust multi-array average normalisation was carried out using R (version3.2.3) software (https://www.r-project.org/). These microarray data were deposited in NCBI’s Gene Expression Omnibus, accession number GSE115944. A variant of Gene Set Enrichment Analysis, ssGSEA, was performed using R to evaluate the pathway activity with c2.all.v.6.2.format (http://software.broadinstitute.org/gsea/msigdb). Two groups were compared by use of SAM method for statistical analysis as previously described.^21^ Ovarian cancer specimens were obtained from 75 patients who underwent primary surgery at Kyoto University Hospital between 1997 and 2012 and prepared for gene expression microarray analysis. The data were previously deposited in the Gene Expression Omnibus (accession Nos: GSE 39204 and GSE55512).^7^

### Membrane-based cytokine array

HM-1 subcutaneous tumours were collected after treatment with anti-VEGF or rat IgG control. Tumour lysates were extracted, and Membrane based cytokine array (R&D, Minneapolis, USA, #ARY006) was performed by following the manufacturer's protocol. Spot intensities were quantified by Image Lab 2.0 software (Bio-Rad, Hercules, USA). Protein expression value was normalised with signal intensities of β-actin in the same membrane.

### ELISA

GM-CSF levels in mouse serum and tumour ascites were measured using a Mouse GM-CSF ELISA MAX Standard according to the manufacturer's instruction (BioLegend, San Diego, USA).

### Reverse transcription (RT)-PCR and real-time quantitative PCR

The total RNA was extracted from cells and frozen tissues using the RNeasy Mini Kit (QIAGEN, Venlo, The Netherlands). Transcriptor High Fidelity cDNA Synthesis Kit was used for cDNA synthesis (Roche Diagnostics, Roswell, GA, USA). For RT-PCR, the cDNAs were amplified using a C1000 Touch^TM^ thermal cycler (Bio-Rad, Hercules, USA). For real-time quantitative PCR, amplification of the target genes and *Gapdh* mRNAs was performed using a Light Cycler 480-II (Roche Diagnostics, Roswell, GA, USA). Relative target gene expression was estimated by dividing the threshold cycle (CT) value of the target gene by the *GAPDH* CT values.

### RelA silencing

RelA-specific siRNA (FlexiTube siRNA Qiagen, catalogue no. 1027416), and negative control siRNA (AllStars Negative Control siRNA, Qiagen) were transfected into cell lines using HiPerFect Transfection Reagent (Qiagen) as previously described.^22^

### Immunoblotting

Cell pellets were lysed with 1 × RIPA buffer (Thermo Fisher Scientific, Waltham, USA) containing a protease inhibitor cocktail (Nacalai Tesque, Kyoto, Japan) and a phosphatase inhibitor cocktail (Nacalai Tesque). Nuclear proteins were collected using NE-PER Nuclear and Cytoplasmic Extraction Reagents (Thermo Fisher Scientific) according to the manufacturer’s protocol. Proteins were subsequently separated by SDS-PAGE and transferred to nitrocellulose membranes. Membranes were immunoblotted with the following antibodies: Rabbit anti-NF-κB p65 (phospho S536) antibody (ab28856, 1:500 dilution, Abcam), rabbit anti-NF-κB p65 antibody (#8242, 1:1000 dilution, Cell Signaling), rabbit anti-GM-CSF antibody (ab9741, 1:2000 dilution, Abcam), rabbit anti-B-actin antibody (#4970, 1:2000 dilution, Cell Signaling) and rabbit anti-HDAC1 antibody (ab109411, 1:1000 dilution, Abcam). The bands were visualised using Molecular Imager Gel DocTMXR + and ChemiDocTMXRS + Systems with Image Lab 2.0 software (Bio-Rad).

### Chemotaxis assay

MDSC in vitro migration was evaluated in 24-well plates with transwell polycarbonate-permeable supports (8.0 μm) (Costar Corning, Cambridge, MA, USA). MDSCs (1.0 × 10^5^; > 90% Gr1 + CD11b + ) were plated in 100 μL of MEMα in the upper compartment 30 min after incubation with anti-GM-CSFRα Ab (10 μg/ml, R&D, Minneapolis, MN, USA), or IgG control Ab, and 500 μL of chemoattractant (ID8-Vegf cell or HM-1 cell tumour-conditioned media (TCM)) was added to the lower compartment. Plates were incubated at 37 °C with 5% CO_2_ for 3 h, and the number of MDSCs in the bottom compartment was counted using CountBright™ Absolute Counting Beads (Life Technology, Carlsbad, CA, USA). To obtain TCM, supernatants were collected from confluent cultures of HM-1 or ID8-Vegf cells cultured in MEMα or RPMI 1640 containing 10% serum.

### Myeloid cell generation assay

To generate in vitro-induced myeloid cells, bone marrow cells were harvested from the femurs and tibias of naive B6 mice. CD11b + population was isolated using auto magnetic-activated cell sorting (autoMACS Pro separator, Miltenyi Biotech). CD11b + cells (1 × 10^5^) were cultured in RPMI1640 supplemented with 10% FBS, 10 ng/mL GM-CSF and 50 μM mercaptoethanol in the absence or presence of 30% v/v tumour supernatant from confluent ID8-Vegf cells in six-well plates. The cultures were maintained at 37 °C in 5% CO_2_ for 5 days. The medium containing each substance was replaced on day 3, and the cells were collected on day 5 for flow cytometric analysis.

### MDSC suppression assay

The in vitro-induced myeloid cell population was collected using Detachin Cell detachment solution (Genlantis, San Diego, USA). Carboxyfluorescein succinimidyl ester (CFSE) (10 μM) was added to the cell suspension (1 × 10^7^ cells/mL) of T cells separated from splenocytes of a wild-type C57BL/6 mouse using mouse Pan T-cell isolation kit (Miltenyi Biotech). Myeloid cells were added to CFSE-labelled T cells at different ratios, harvested in 96-well plates and activated with Dynabeads Mouse T-activator CD3/28 (VERITAS, Tokyo, Japan) for 72 h at 37 °C. T-cell proliferation was evaluated with flow cytometry.

### Immunohistochemical analysis of an ovarian cancer case

Surgical specimens from patients with endometrioid ovarian cancer who underwent primary surgery at Kindai University Hospital were collected after approval of the study protocol by the Institutional Ethical Committee. We selected a recurrence case after the combination therapy of paclitaxel, carboplatin and bevacizumab. The relevant clinical data were collected by retrospective review of patient files. Immunostaining was performed using the streptavidin–biotin–peroxidase method as previously described.^6^ For HIF1α, GM-CSF, NF-kB, CD33 and CD8, the samples were incubated with rabbit anti-HIF1α polyclonal antibodies (Abs, ab46154, 1:200 dilution, Abcam, Cambridge, UK), rabbit anti-GM-CSF Abs (clone SP35, 1:100 dilution, Cell-Marque, Rocklin, CA, USA), rabbit anti-NF-κB polyclonal antibody (clone Phospho S536, 1:200 dilution, Abcam, Cambridge, UK), mouse anti-CD33 monoclonal Abs (clone PWS44, 1:200 dilution, Leica Biosystems, Nussloch, Germany) and mouse anti-CD8 Abs (clone C8/144B, 1:100 dilution, Nichirei Biosciences, Tokyo, Japan).

### Statistical analysis

For all experiments, quantitative data are shown as average ± SEM from three independent experiments. Group comparisons were performed by using a Mann–Whitney U test. Univariate prognostic analysis was done using a log-rank test. All statistical analyses were performed using Graph Pad Prism 6.0. *P* < 0.05 was considered statistically significant.

## Results

### Anti-VEGF therapy increases MDSC number and reduces CD8 + cell number in tumour

**Fig. 1:**
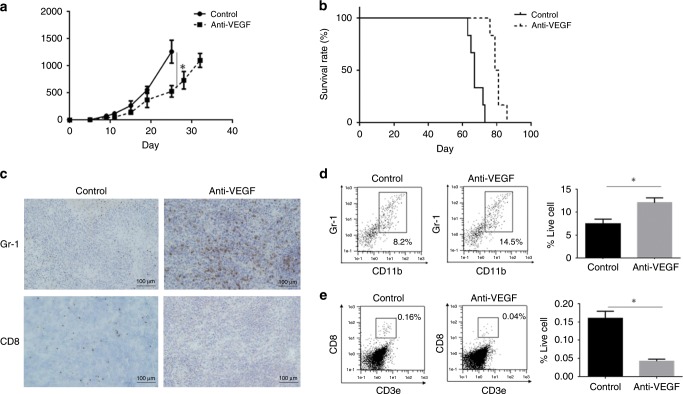
Anti-VEGF therapy induces MDSCs and decreases CD8+lymphocytes in tumour. **a** Growth of HM-1 tumours treated with anti-VEGF antibody (dotted black line) or rat IgG control (solid black line) (*n* = 6, *P < 0.05). Data are represented as mean ± SEM. **b** Survival analysis of ID8-Vegf tumour-bearing mice treated with anti-VEGF antibody (dotted black line) or rat IgG control (solid black line) (*n* = 6, **P < 0.005). **c** Representative immunostaining images of HM-1 treated with or without anti-VEGF antibody for Gr-1 and CD8. Scale bar, 100 μm. **d**, **e** Proportions of CD11b + Gr1 + cells and CD3e + CD8 + cells in HM-1 tumour. *P < 0.05. Data are represented as mean ± SEM.

We treated different mouse ovarian cancer models—HM-1 subcutaneous tumour model, ID8 peritoneal tumour model and ID8-Vegf peritoneal tumour model—with anti-VEGF antibody. We observed that tumour growth was slightly inhibited by anti-VEGF therapy in HM-1 tumour model and ID8-Vegf peritoneal tumour model (Fig. 1a, b). On the other hand, ID8 peritoneal tumour-bearing mice were almost completely cured by anti-VEGF antibody (Supplementary Fig. S1). These results indicate that the efficacy of anti-VEGF therapy is different between tumours. Previously, we have shown that VEGF expression in ovarian cancer induces MDSCs and promotes tumour progression through immunosuppression.^9^ Contrary to expectations, in anti-VEGF antibody-treated HM-1 tumour, MDSC numbers were increased, whereas CD8 + lymphocyte numbers were decreased (Fig. 1c–e). These results suggest that anti-VEGF therapy alters the tumour microenvironment, and compensatory signals induce resistance to anti-VEGF therapy through induction of MDSCs in the tumour.

### Hypoxia induced by anti-VEGF therapy recruits MDSCs

**Fig. 2:**
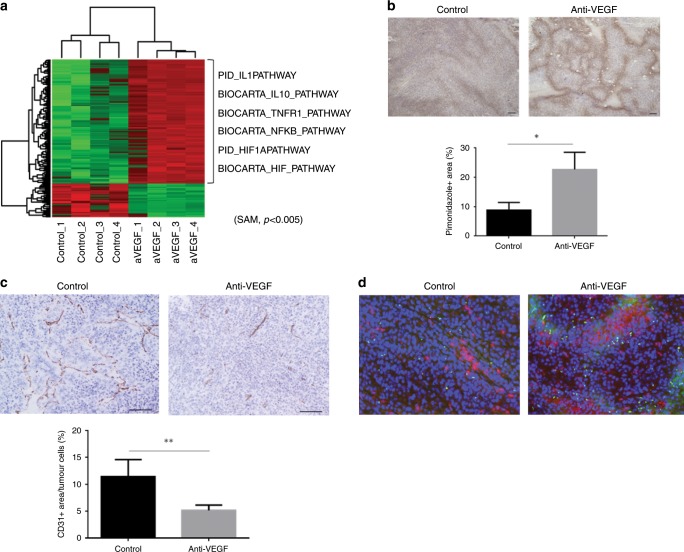
Anti-VEGF therapy induces tumour hypoxia and attracts MDSCs. **a** Pathway analysis based on gene expression microarray of HM-1 tumours treated with anti-VEGF antibody or control (*n* = 4, *P < 0.005). Heatmap shows different pathway scores calculated by ssGSEA. **b** Immunostaining of HM-1 tumours with hypoxic probe, pimonidazole. Scale bar, 100 μm. Percentages of pimonidazole + area in tumour cells were analysed (*P < 0.05). Data are represented as mean ± SEM, *n* = 5 per group. **c** Immunostaining of HM-1 tumours for CD31. Scale bar, 100 μm. Percentages of CD31 + area in tumour cells were analysed (*P < 0.05). Data are represented as mean ± SEM, *n* = 5 per group. **d** Immunofluorescence staining of HM-1 tumours for pimonidazole and Gr-1. Green shows pimonidazole-positive cells, red shows Gr-1-positive cells and blue shows 4′,6-diamidino-2-phenylindole (DAPI)-stained nuclei.

To identify varying signals in HM-1 tumour after anti-VEGF therapy, we performed pathway analysis based on a gene expression microarray (Supplementary Table S1). HIF-1α-related hypoxic pathways, NF-κB signalling pathways and cytokine-related pathways were upregulated in tumour treated with anti-VEGF antibody (Fig. 2a). Immunohistochemical analysis showed that anti-VEGF therapy decreased the number of tumour vessels and increased the hypoxic area in the tumour microenvironment (Fig. 2b, c). Moreover, Gr-1 + MDSCs were found to be preferentially enriched in the hypoxic area (Fig. 2d). These results suggest that MDSCs were chemoattracted by some signals from hypoxic tumour cells induced by anti-VEGF therapy.

### GM-CSF expression in tumour microenvironment is upregulated after anti-VEGF therapy

**Fig. 3:**
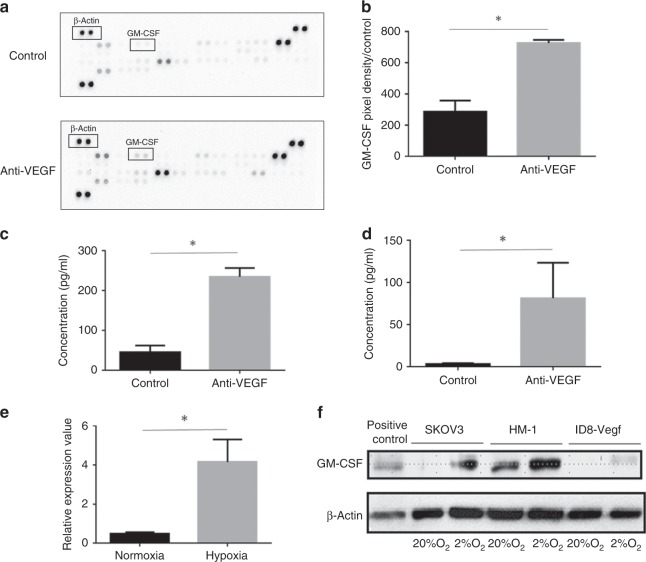
Expression of GM-CSF is upregulated in a-VEGF abs-treated tumour. **a** The representative pictures of membrane cytokine array of HM-1 tumour lysates treated with anti-VEGF antibody or control. **b** GM-CSF expression values of HM-1 tumour lysates based on membrane array analysis (*n* = 4, *P < 0.05). Each spot density was normalised with densities of β-actin. Data are represented as mean ± SEM. **c** GM-CSF protein levels in serum of HM-1 tumour-bearing mice treated with anti-VEGF antibody or control (*n* = 6, *P < 0.05). Data are represented as mean ± SEM. **d** GM-CSF protein concentrations in ascites of ID8-Vegf tumour-bearing mice treated with anti-VEGF antibody or control (*n* = 6, *P < 0.05). Data are represented as mean ± SEM. **e** Quantitative PCR analysis of Gm-csf gene expression in HM-1 cells cultured under normoxic or hypoxic conditions. Data are represented as mean ± SEM, *n* = 5 per group. **f** GM-CSF protein expression in HM-1, ID8-Vegf and SKOV3 cells cultured under normoxic or hypoxic conditions.

To identify which soluble factors, including cytokines and growth factors, are altered by anti-VEGF therapy, we performed membrane-based protein array analysis using HM-1 tumour lysates. We noticed that GM-CSF was one of the proteins with the maximum changes in expression levels (Fig. 3a, b; Supplementary Table S2). Serum GM-CSF levels were also higher in anti-VEGF antibody-treated mice (Fig. 3c). In addition, in ID8-Vegf peritoneal dissemination mouse model, GM-CSF levels in the tumour ascites were higher in the anti-VEGF antibody-treated group (Fig. 3d), indicating that anti-VEGF therapy enhances GM-CSF levels in the tumour microenvironment.

### GM-CSF is upregulated under hypoxic conditions through the NF-κB pathway

**Fig. 4:**
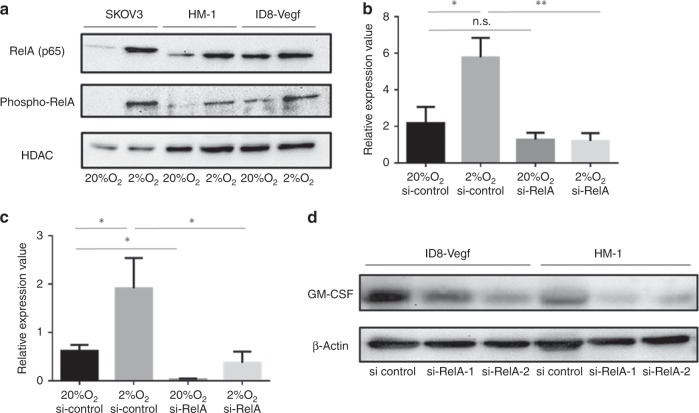
Upregulation of GM-CSF expression under hypoxia is mediated via the NF-κB pathway. **a** Protein expression of RelA (p65), a component of NF-κB in HM-1, ID8-Vegf and SKOV3 cells under normoxic or hypoxic conditions. **b** Quantitative PCR analysis of Gm-csf gene expression in HM-1 cells cultured under normoxic or hypoxic conditions with NF-κB gene silencing (*P < 0.05, **P < 0.01). Data are represented as mean ± SEM, *n* = 5 per group. **c** Quantitative PCR analysis of Gm-csf gene expression in ID8-Vegf cells cultured under normoxic or hypoxic conditions with NF-κB gene silencing (*P < 0.05). Data are represented as mean ± SEM, *n* = 5 per group. **d** GM-CSF protein expression in HM-1 and ID8-Vegf cells cultured under hypoxic conditions with NF-κB gene silencing.

To identify the mechanism of GM-CSF upregulation after anti-VEGF treatment, mouse ovarian cancer HM-1 and ID8-Vegf cells and human ovarian cancer SKOV3 cells were cultured under hypoxic conditions. GM-CSF expression, along with VEGF-A and Cox 2 levels, was upregulated under hypoxic conditions (Fig. 3e, f; Supplementary Fig. S2a) GM-CSF protein levels were also found to be higher in human ovarian cancer cells under hypoxic conditions (Fig. 3f). GM-CSF expression was not upregulated and cultured with CoCl_2_, which activates the HIF1-α signalling pathway (Supplementary Fig. S2b). We then examined the changes in NF-κB signalling activity, which regulates expression of various cytokines in ovarian cancer cells under hypoxic conditions. Expression levels of NF-κB subunits in ovarian cancer cells were upregulated under hypoxic conditions (Fig. 4a). We next examined if GM-CSF upregulation under hypoxic conditions was mediated through activation of NF-κB signalling. Silencing NF-κB expression inhibited the upregulation of GM-CSF in hypoxic conditions (Fig. 4b–d), suggesting that hypoxic tumour cells upregulate GM-CSF expression through the activation of NF-κB signalling.

### GM-CSF expression in tumour microenvironment induces immunosuppressive MDSCs

**Fig. 5:**
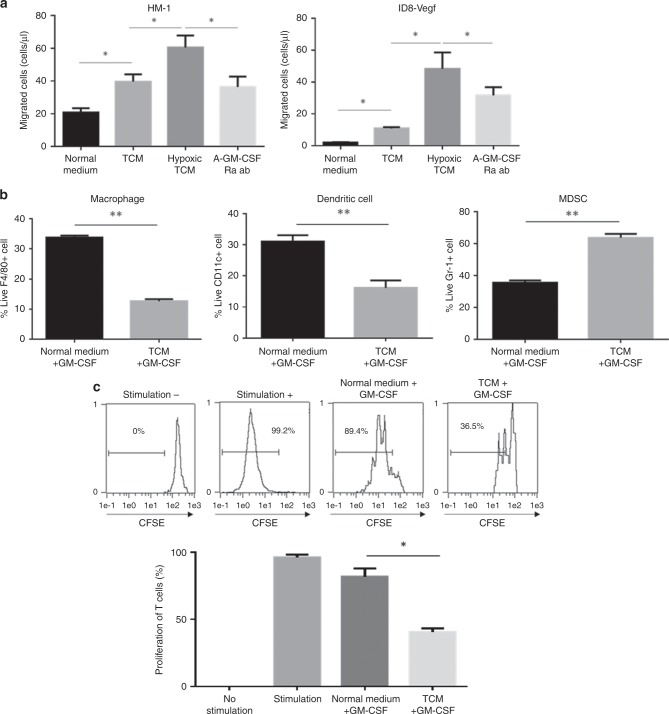
The impact of GM-CSF signals on MDSC migration and differentiation. **a** The number of migrated Gr-1 + cells in response to HM-1 or ID8-Vegf cell-conditioned medium cultured under normoxic or hypoxic conditions. Gr-1 + cells were treated with rat IgG control or anti-GM-CSFRa antibody *P < 0.05. Data are represented as mean ± SEM, *n* = 5 per group. **b** Flow cytometric analysis for the presence of TAM, DC and MDSCs induced by in vitro generation assay. CD11b + cells were cultured in the presence of GM-CSF with or without ID8-Vegf cell conditioned medium. **P < 0.005. Data are represented as mean ± SEM, *n* = 5 per group. **c** The proportions of proliferation of CD8 + T cells after co-culture with in vitro-induced myeloid cells. The histogram shows the percentages of proliferated T cells. *P < 0.05. Data are represented as mean ± SEM. *n* = 4 per group.

To examine the effect of GM-CSF on migration of MDSCs in tumour microenvironment, we performed chemotaxis assay using HM-1 and ID8-Vegf cell tumour supernatants, respectively. We observed an increase in the number of migrating MDSCs towards hypoxic tumour cell supernatants, and an inhibition of GM-CSF signalling suppressed this migration of MDSCs (Fig. 5a). GM-CSF is reported to be an essential factor for myeloid cell differentiation and maintenance in the bone marrow.^13^ We performed an ex vivo MDSC generation assay to examine the impact of GM-CSF on differentiation of MDSCs in tumour microenvironment. CD11b + myeloid cells differentiated into macrophages and dendritic cells in the absence of tumour cell supernatant, whereas CD11b + myeloid cells differentiated into MDSCs upon supplementation with tumour cell supernatant enriched in GM-CSF (Supplementary Fig. S3; Fig. 5b). Myeloid cells generated in the medium containing tumour cell supernatant suppressed proliferation of CD8 + T cells (Fig. 5c). These results indicate that GM-CSF in the tumour microenvironment promoted the myeloid cell differentiation into immunosuppressive MDSCs.

### Anti-GM-CSF antibody therapy enhances the efficacy of anti-VEGF therapy by reducing MDSCs

**Fig. 6:**
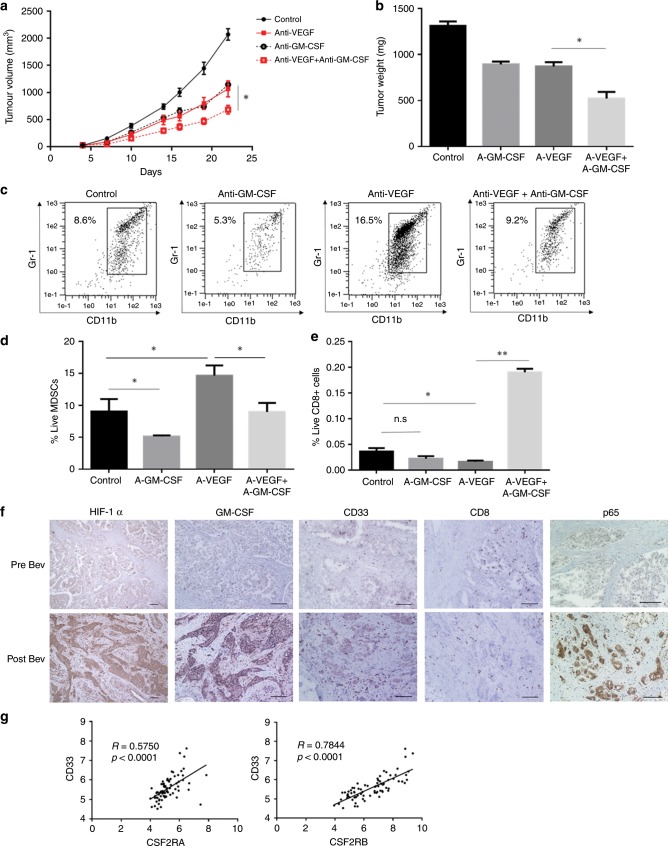
A-GM-CSF abs enhances the efficacy of a-VEGF abs against HM-1 tumours via lymphocyte activation. **a** Growth curves of HM-1 tumours in B6C3F1 mice under different treatments as indicated: black solid line indicates rat IgG control. Black dotted line indicates anti-VEGF antibody. Red solid line indicates anti-GM-CSF antibody. Red dotted line indicates a combination of anti-VEGF antibody and anti-GM-CSF antibody (*n* = 6, *P < 0.05). Data are represented as mean ± SEM. **b** Tumour weight of HM-1 tumours treated with different treatments (*n* = 6, *P < 0.05). Data are represented as mean ± SEM. **c** The representative figures of dot plots for MDSCs in HM-1 tumour in different treatment groups. **d** The frequencies of CD11b + Gr1 + cells in HM-1 tumour to tumour live cells (*n* = 6, *P < 0.05). Data are represented as mean ± SEM. **e** The frequencies of CD8 + cells to tumour live cells in HM-1 tumour treated with a-VEGF abs and/or a-GM-CSF abs (*n* = 6, *P < 0.05). Data are represented as mean ± SEM. **f** The representative images of immunohistochemistry of ovarian cancer clinical samples for HIF1a, GM-CSF, NF-κB, CD33 and CD8. The case received combination therapy of paclitaxel, carboplatin and bevacizumab after probe laparotomy. Resistant tumours against bevacizumab therapy were secondary resected. Scale bar, 100 μm. **g** The correlation analysis of expression values of CD33 and CSF2 receptors in ovarian cancer samples in Kyoto University (*n* = 74).

To examine whether the inhibition of GM-CSF signalling could enhance the efficacy of anti-VEGF therapy, we treated mice with a combination of anti-VEGF antibody and anti-GM-CSF antibody. Anti-GM-CSF antibody monotherapy was moderately effective; however, the addition of anti-GM-CSF antibody enhanced the therapeutic efficacy of anti-VEGF antibody (Fig. 6a, b). As a result of the combination therapy, MDSC numbers in the tumour were significantly decreased (Fig. 6c, d), whereas CD8 + T-cell numbers in the tumour showed an increase (Fig. 6e). We analysed the percentage of granulocytic MDSCs (G-MDSCs) and monocytic MDSCs (M-MDSCs), which are subpopulations of MDSCs, upon a combined treatment, and found that both of these populations showed a marked reduction, when compared with the anti-VEGF treatment alone (Supplementary Fig. S4). We also tried a combination therapy of anti-CXCR2 antagonist and anti-VEGF antibody, since CXCR2 ligand, CXCL2 is a known chemoattractant for MDSCs, and its upregulation was observed in the protein array (Supplementary Table S2). Although Anti-CXCR2 antagonist monotherapy showed moderate efficacy, anti-CXCR2 antagonists in combination with anti-VEGF antibody did not show a synergistic enhancement in the therapeutic efficiency (Supplementary Fig. S5a). We treated lymphocyte-deficient mice with the combination of anti-VEGF and anti-GM-CSF antibodies, and observed no discernible change in the efficiency of therapy in comparison with only anti-VEGF antibody treatment (Supplementary Fig. S5b). These results suggest that the resistance to anti-VEGF therapy is associated with GM-CSF-mediated expansion of MDSCs resulting in lymphocyte suppression.

### Bevacizumab induces GM-CSF and MDSCs in clinical setting

We examined GM-CSF expression and MDSC infiltration in a pair of clinical ovarian cancer tumour samples before and after bevacizumab treatment from the same patient. The case was clinically refractory to bevacizumab treatment. HIF1-α, NF-κB and GM-CSF expression levels in tumour were higher, and CD33 + MDSCs showed an increased retention in the tumour stroma, whereas CD8 + lymphocyte numbers in tumour epithelium were markedly reduced after bevacizumab treatment (Fig. 6f). In both ovarian cancer data sets of our samples^7^ and The Cancer Genome Atlas (TCGA),^23^ expression of CD33 was positively correlated with expression of CSF2RA/B that is the receptor for GM-CSF (Fig. 6g; Supplementary Fig. S6a), indicating that GM-CSF could be an inducer of MDSCs. We analysed another expression microarray data set of a glioma xenograft that was treated with bevacizumab.^24^ The bevacizumab-treated set showed an upregulation in the expression of GM-CSF, and genes in hypoxia, NF-κB and myeloid cell maturation signalling pathways (Supplementary Fig. S6b), indicating that similar signalling also occurs in other tumour types.

## Discussion

Effective ovarian cancer treatment requires overcoming the resistance to available therapeutic options such as bevacizumab and also increasing the sensitivity of tumours to the same. Ovarian cancer is classified into four molecular subtypes on the basis of gene expression profiles as mesenchymal, proliferative, differentiated and immunoreactive, respectively.^22^ It has been reported that patients’ prognoses and sensitivity to drugs were different between these subtypes.^25^ Recently, Kommoss S et al. showed that bevacizumab treatment was beneficial in proliferative and mesenchymal cancers, but not for immunoreactive cases.^26^ These reports indicate that immune signalling in tumours contributes to bevacizumab resistance. In this study, the efficacy of anti-VEGF antibody was found to be different between ID8 and ID8-Vegf tumours (Supplementary Fig. S1). Previously, we reported that infiltrating MDSC numbers were increased in ID8-Vegf tumour as compared with those in ID8 tumour, which ultimately leads to immunosuppression.^9^ Tumour-infiltrating Gr-1 + MDSCs are reported to contribute to the resistance to anti-VEGF therapy in another tumour model.^27^ Taken together, these results indicate that immunosuppression caused by MDSCs is potentially associated with resistance to anti-VEGF antibody.

VEGF is an angiogenic as well as an immunosuppressive factor, which inhibits the maturation of dendritic cells and lymphocyte activity.^28^ Previously, we showed that VEGF signalling directed MDSCs into the tumour site.^9^ We hypothesised that anti-VEGF therapy reduced MDSC localisation to tumours as previously reported.^29^ However, experimental evidence showed that infiltrating MDSC numbers showed a marked increase, and CD8 + lymphocyte numbers showed a significant reduction in tumour sites after anti-VEGF antibody treatment in this study, in both murine models and clinical samples (Fig. 1c, d, 6f). One of the likely causes could be the fact that anti-VEGF therapy induces disruption of tumour vessels and consequent hypoxia, which results in dramatic changes in various signalling cascades in the tumour microenvironment, as seen in microarray analysis (Fig. 2a). Similar variations in gene expression were also observed in glioma xenografts (Supplementary Fig. S6b). Together, these results indicate that immunosuppression caused by hypoxia is the key to anti-VEGF resistance.

Anti-VEGF therapy and subsequent hypoxia induced GM-CSF expression (Fig. 2b–d), which promoted the differentiation and migration of MDSCs in the tumour microenvironment (Fig. 5a, b). GM-CSF has been reported as an immune activator that maintains the function of dendritic cells.^30^ On the other hand, reports have shown that GM-CSF inhibits lymphocyte activity through induction of MDSCs.^31,32^ We show that GM-CSF can potentiate the differentiation of myeloid cells into dendritic cells or MDSCs in the absence and presence of tumour supernatant, respectively (Fig. 5b). Morales J. K. et al. reported that GM-CSF in tumour supernatants of mammary tumour cell line (MMC cells), promoted the generation of MDSCs.^33^ Further investigation is required to identify the key factor to polarise undifferentiated myeloid cells towards MDSCs.

Expression of GM-CSF in ovarian cancer cell lines was upregulated via NF-κB signalling under hypoxic conditions. Although HIF-1α is the major transcriptional factor for several genes under hypoxic conditions, GM-CSF in ovarian cancer cell was not upregulated after treatment with CoCl_2_, which mimics hypoxia-stabilising HIF-1α protein (Supplementary Fig. S2b). Therefore, we believe that HIF-1α does not contribute to the regulation of GM-CSF expression. A report showed that under hypoxic conditions, renal cell carcinoma cell lines induced the TNF-α signalling pathway via GM-CSF, CXCL2, CXCL3 and PTGS2.^34^ Interestingly, these signalling factors were also observed to be upregulated in the cytokine array after anti-VEGF treatment in this study. NF-κB subunits have been reported to bind to the promoter of GM-CSF,^35^ which supports our hypothesis that GM-CSF is regulated through the NF-κB pathway.

In addition to GM-CSF, anti-VEGF therapy induced expression changes in the levels of several cytokines such as CXCL2 and CCL2, which have been reported to be the chemoattractants for myeloid cells. We have shown that upregulation of CXCL2 expression through NF-kB signalling in ovarian cancer induces differentiation of MDSCs and immunosuppression.^36^ CXCL2 expression in ovarian cancer cells was also upregulated under hypoxic conditions; hence, we hypothesised that CXCL2 signal could be a key inducer of MDSCs following anti-VEGF treatment. However, a blockade of CXCL2 signal could not improve the efficacy of anti-VEGF antibody treatment (Supplementary Fig. S5a). CXCL2 is a specific inducer of Ly6G high granulocytic MDSCs (G-MDSCs), but not of Ly6C high monocytic MDSCs (M-MDSCs). Several reports showed that both of these populations possess immunosuppressive ability,^37,38^ and here, we show that both populations showed an increase in percentage after anti-VEGF treatment (Supplementary Fig. S4). Since G-MDSCs are differentiated from M-MDSCs,^39^ we assume that targeting only G-MDSCs is not enough to improve the lymphocyte activity in tumour sites. Cytokines such as GM-CSF, CCL2 and CCL3, which are upregulated upon anti-VEGF treatment, are reported to act as inducers of dendritic cells. Several reports have shown that tumour-associated macrophage (TAM) is associated with resistance with bevacizumab therapy.^40,41^ In this study, we have analysed the numbers of CD11c + dendritic cells (DCs) and CD206 + F4/80 + TAMs after anti-VEGF treatment; however, there was no significant change in the population of DCs or TAMs (Supplementary Fig. S4). Taken together, M-MDSCs play a major role in immunosuppression after anti-VEGF therapy. MDSC counts or upregulation of GM-CSF and the related chemokines could be used as biomarkers of bevacizumab resistance, but the issue requires further investigation in clinical samples.

Anti-GM-CSF monotherapy was slightly effective in ovarian cancers, and the combination of anti-GM-CSF antibody with anti-VEGF antibody was found to be highly effective in this study (Fig. 6a, b). Although GM-CSF is reported to be a growth factor of tumour cells,^42^ we compared the proliferation of GM-CSF-depleted cells (HM-1 sh-Csf2 cells) with control cells by proliferation assay to find that there was no difference between growth of these cells (data not shown). GM-CSF might not directly affect the growth of ovarian cancer cells in this experiment. Constitutive expression of GM-CSF in the components of HM-1 tumour tissue might induce tumour-promoting MDSCs. The combination of anti-VEGF antibody with anti-GM-CSF antibody decreased MDSC count, and increased CD8 + cell number, indicating that anti-GM-CSF antibody improves tumour immunity. In a lymphocyte-deficient murine model, anti-GM-CSF antibody did not improve the efficacy of anti-VEGF antibody treatment, which verifies that GM-CSF behaves as an immunosuppressive factor. Together, the above data indicate that targeting GM-CSF or MDSCs has the potential to reverse resistance to anti-VEGF therapy.

Furthermore, upregulation of GM-CSF after anti-VEGF therapy was confirmed in resistant tumours. The change in GM-CSF protein level after bevacizumab therapy could be a predictive biomarker.

In conclusion, we showed that anti-VEGF antibody treatment induces tumour hypoxia, which increases the expression of GM-CSF in tumour cells. GM-CSF recruits and maintains MDSCs in tumour microenvironment and suppresses antitumour immunity, which causes resistance to anti-VEGF antibody. Treatment targeting MDSCs or GM-CSF could be a promising combination candidate to improve the efficacy of anti-VEGF antibody treatment.

## Supplementary information

Supplementary data

## Data Availability

Microarray datasets for this work are accessible on Gene Expression Omnibus website. (https://www.ncbi.nlm.nih.gov/geo/query/acc.cgi?acc=GSE115944).
