# Supplementary material for: Anti-VEGF therapy resistance in ovarian cancer is caused by GM-CSF-induced myeloid-derived suppressor cell recruitment
Source: Br J Cancer. 2020 Jan 14;122(6):778–88. doi: 10.1038/s41416-019-0725-x (PMC7078258; doi:10.1038/s41416-019-0725-x)
Supplement: Supplementary file 1 — Supplementary data [file 41416_2019_725_MOESM1_ESM.docx]

**Supplementary Figures**


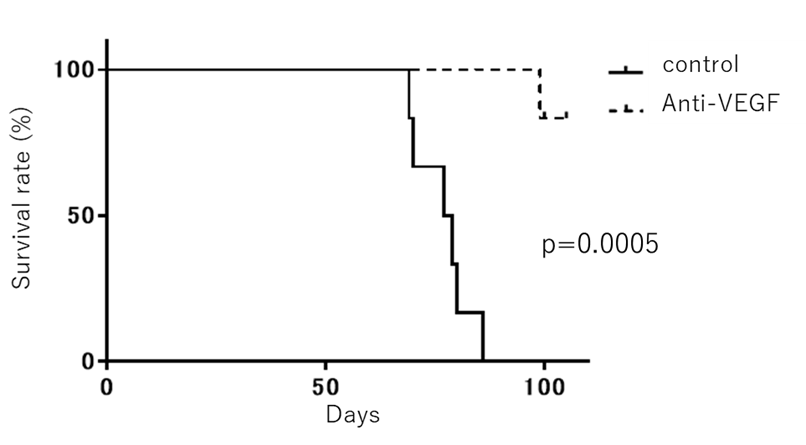


**Supplementary Figure S1.**

Survival analysis of C57/BL6 mice bearing ID8-GFP peritoneal tumor treated with anti-VEGF antibody or Rat IgG control(n=6). **P<0.005.

**
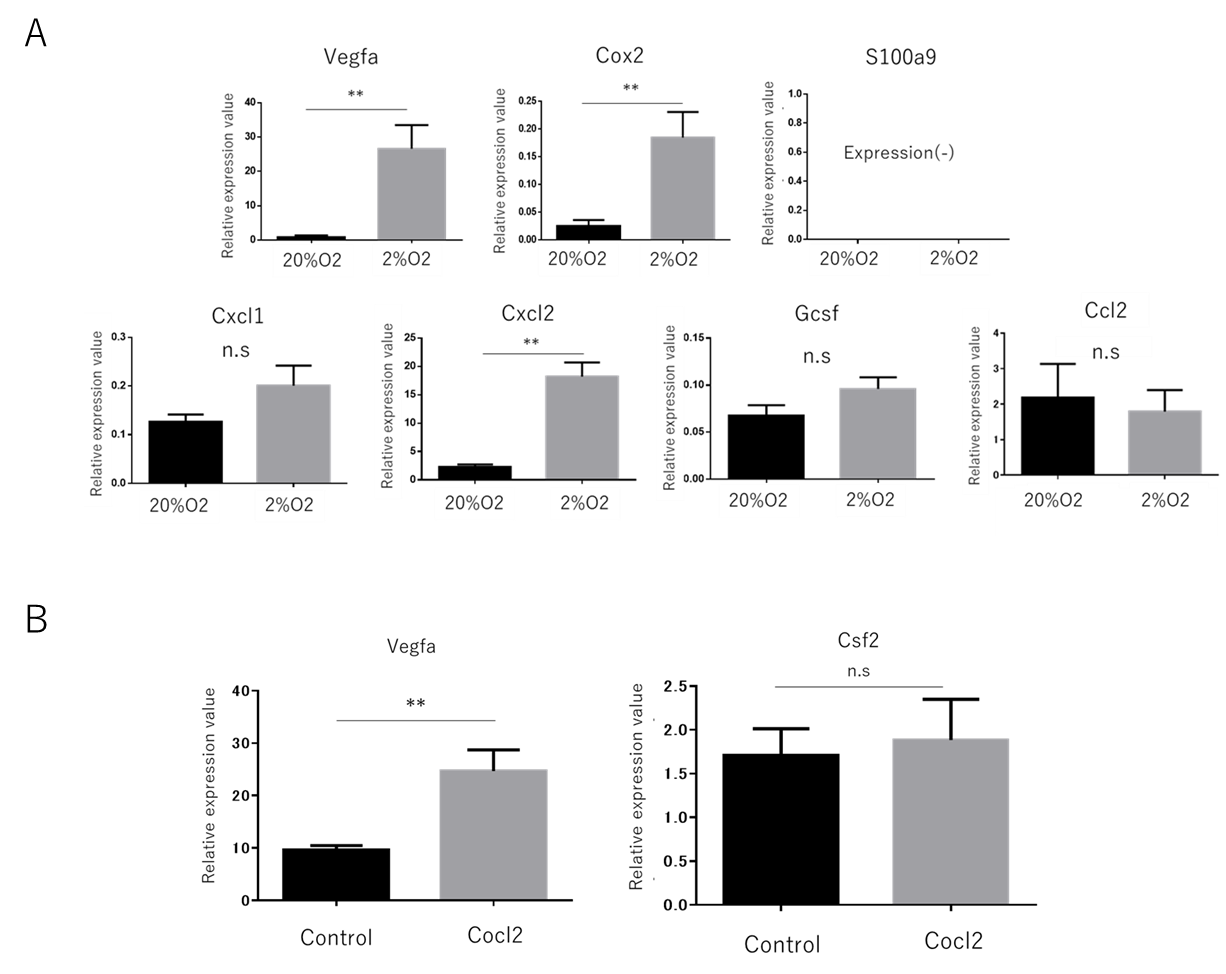
**

**Supplementary Figure S2.**

1. Quantitative PCR analysis of HM-1 cells cultured under normoxic condition(20%O2) or hypoxic condition(2%O2). Data are represented as mean ± SE (n=5)　*p<0.05 **p<0.005.

(B) Quantitative PCR analysis of HM-1 cells cultured in the medium supplemented with or without Cocl2 solution. Data are represented as mean ± SE (n=5)　**p<0.005.


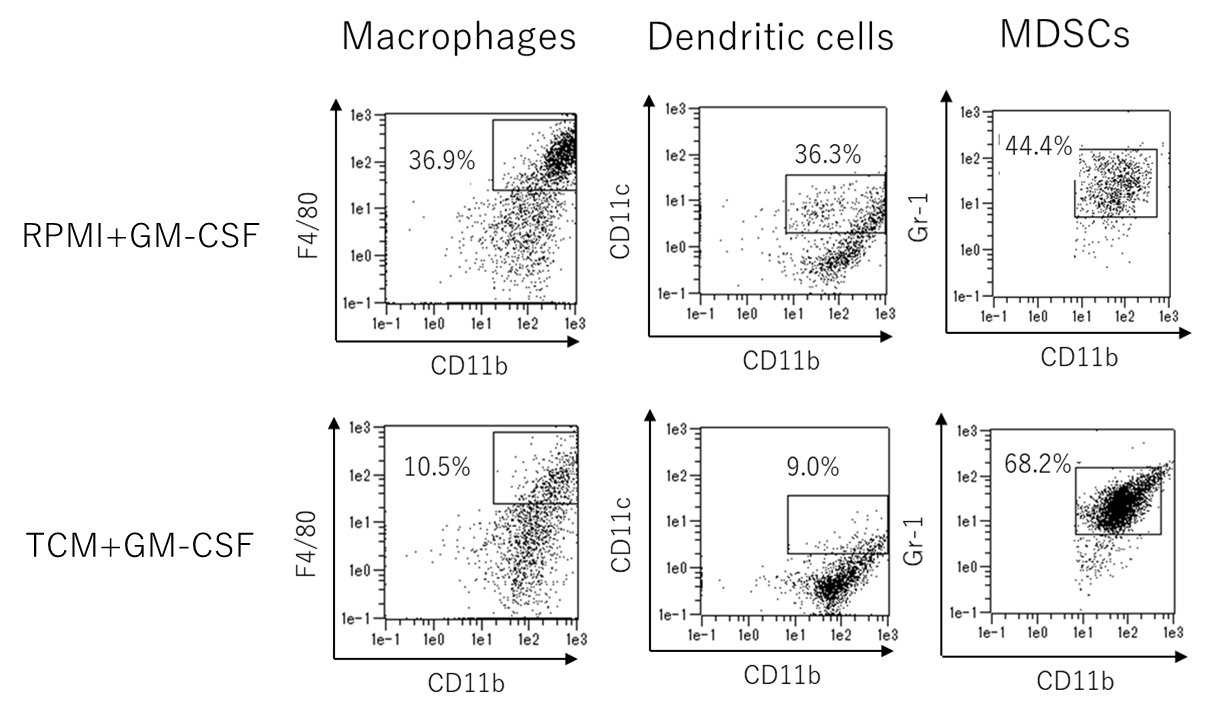


**Supplementary Figure S3.**

Flow cytometric analysis of in-vitro induced myeloid cell populations. Total myeloid cells were gated for CD45+. CD11b+F4/80+ cells indicate Macrophages. CD11b+ CD11c+ cells indicate Dendritic cells. CD11b+ Gr-1+ populations indicate MDSCs.


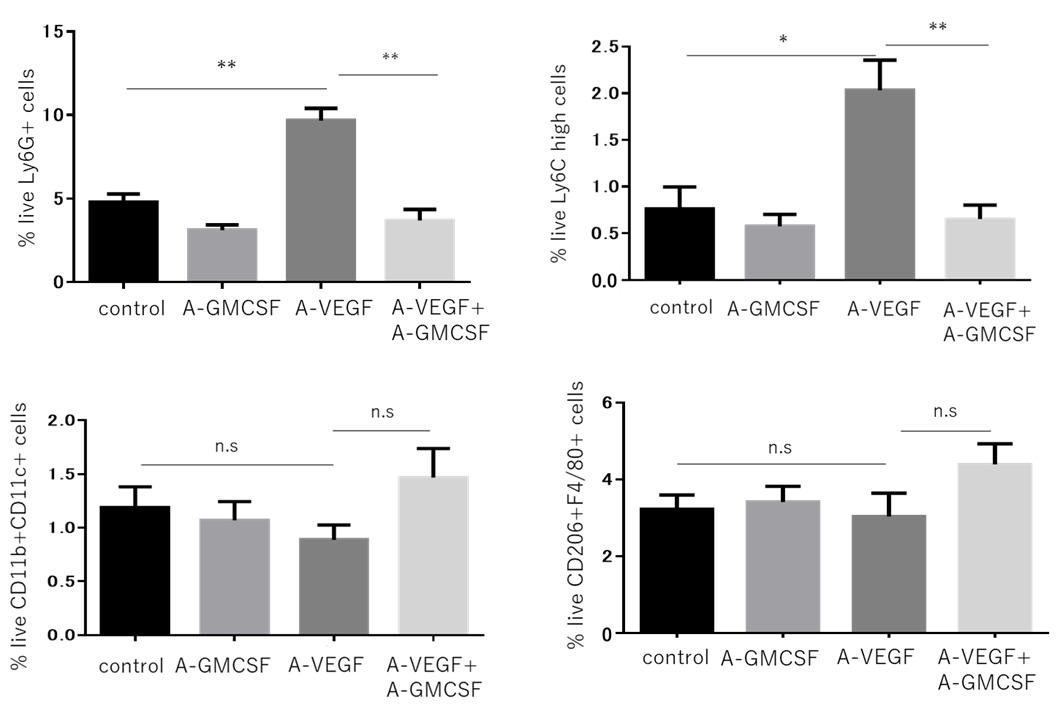


**Supplementary Figure S4.**

Flow cytometric analysis of HM-1 tumors treated with alone or combination of anti-VEGF antibody and anti-GM-CSF antibody. Data are represented as mean ± SE (n=6). *p<0.05 **p<0.005. Data are represented as mean ± SE (n=6)


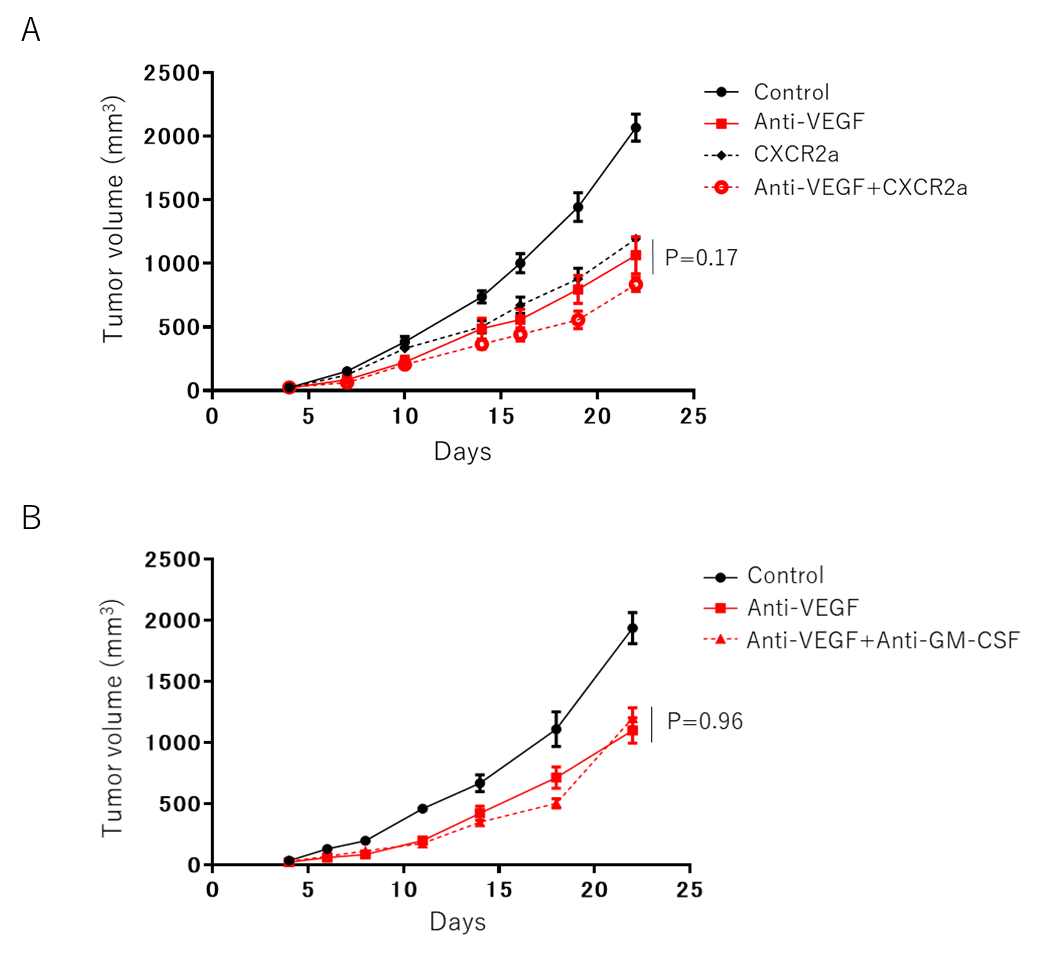


**Supplementary Figure S5.**

(A)The growth curve of HM-1 subcutaneous tumors under different treatments as indicated: control (Rat IgG), aVEGF(5mg/kg), CXCR2a (2mg/kg), combination of anti-VEGF antibody and CXCR2 antagonist. Data are represented as mean ± SE (n=6)

(B) Growth curves of HM-1 tumors in CD-1 Nude mice under different treatments as indicated: control (Rat IgG), anti-VEGF, anti-VEGF+anti-GM-CSF. Data are represented as mean ± SEM (n=6).


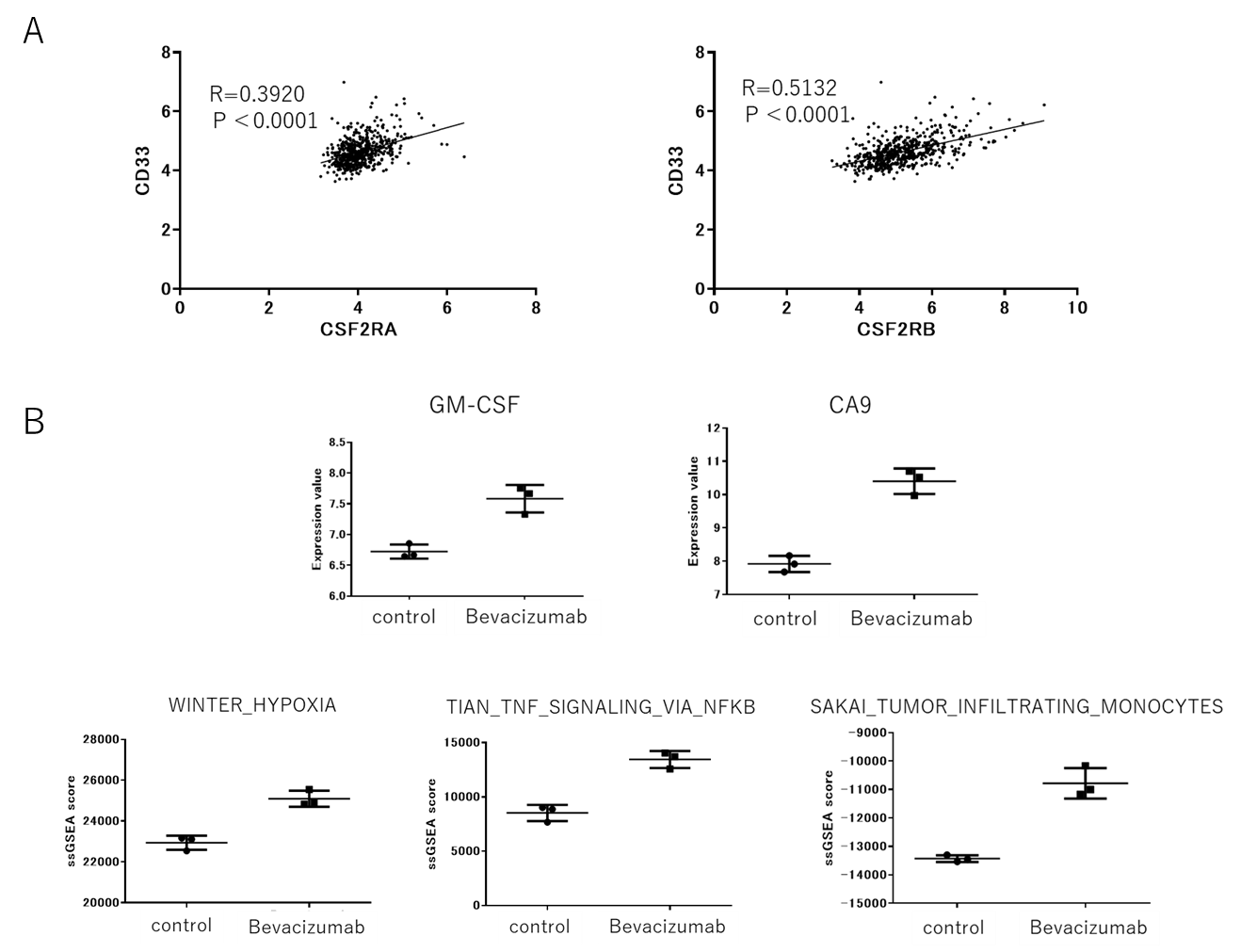


**Supplementary Figure S6.**

(A) Correlation analysis of gene expression values of CD33 and CSF2 receptors in ovarian cancer tissues in TCGA dataset.

(B) Analysis of gene expression microarray of glioma xenografts treated with or without

Bevacizumab deposited at GSE37956. Data are represented as mean ± SE (n=3)

**Supplementary Table S1.**

The list of pathway scores of HM-1 tumors treated with anti-VEGF antibody or Rat IgG control.

**Supplementary Table S2.**

Protein expression profiles of HM-1 tumor lysates treated with anti-VEGF antibody or Rat IgG control.
